# Effects of Nano-MnO_2_ on Dopaminergic Neurons and the Spatial Learning Capability of Rats

**DOI:** 10.3390/ijerph110807918

**Published:** 2014-08-06

**Authors:** Tao Li, Tingting Shi, Xiaobo Li, Shuilin Zeng, Lihong Yin, Yuepu Pu

**Affiliations:** 1 Key Laboratory of Environmental Medicine Engineering, Ministry of Education, School of Public Health, Southeast University, Nanjing 210009, China; E-Mails: llg501@aliyun.com (T.L.); lixiaobo1007@hotmail.com (X.L.); lhyin@seu.edu.cn (L.Y.); 2 Institute of Neurobiology, Southeast University, Nanjing 210009, China; E-Mails: shitingting.student@sina.com (T.S.); zsl@seu.edu.cn (S.Z.)

**Keywords:** nano-MnO_2_, Morris water maze, dopaminergic neurons, glial fibrillary acidic protein

## Abstract

This study aimed to observe the effect of intracerebrally injected nano-MnO_2_ on neurobehavior and the functions of dopaminergic neurons and astrocytes. Nano-MnO_2_, 6-OHDA, and saline (control) were injected in the substantia nigra and the ventral tegmental area of Sprague-Dawley rat brains. The neurobehavior of rats was evaluated by Morris water maze test. Tyrosine hydroxylase (TH), inducible nitric oxide synthase (iNOS) and glial fibrillary acidic protein (GFAP) expressions in rat brain were detected by immunohistochemistry. Results showed that the escape latencies of nano-MnO_2_ treated rat increased significantly compared with control. The number of TH-positive cells decreased, GFAP- and iNOS-positive cells increased significantly in the lesion side of the rat brains compared with the contralateral area in nano-MnO_2_ group. The same tendencies were observed in nano-MnO_2_-injected rat brains compared with control. However, in the the positive control, 6-OHDA group, escape latencies increased, TH-positive cell number decreased significantly compared with nano-MnO_2_ group. The alteration of spatial learning abilities of rats induced by nano-MnO_2_ may be associated with dopaminergic neuronal dysfunction and astrocyte activation.

## 1. Introduction

Manganese (Mn) is an abundant, naturally occurring element in the Earth’s crust. In organisms, Mn is a cofactor of several enzymes, such as transferases, hydrolases, lyases, arginase, glutamine synthetase, and superoxide dismutase; Mn is also found in integrins [1]. Considering the dependence of multiple enzymes on Mn, this element is essential for various physiological processes, such as modulation of the immune system, stellate process production in astrocytes, cell adhesion, and protein and carbohydrate metabolism [2,3,4,5]. Although Mn is essential for metabolic functions, excessive exposure to Mn is a well-recognized occupational hazard. Occupational exposure to Mn for six months to two years can cause an extrapyramidal syndrome, referred to as manganism, which exhibits symptoms similar to idiopathic Parkinson’s disease at molecular and clinical levels [6,7,8].

Nano-sized materials are novel substances rapidly developed and usually measured as 1 to 100 nm size in one dimension. At a nanometer scale, the surface area increases rapidly as particle size decreases, as a result, the electromagnetic, optical, thermal, and mechanical properties of nanomaterials change. With the rapid development of nanotechnology and extensive application of nanomaterials, the health effects of nanoparticles on human body, particularly on the nervous system, have been widely investigated [9,10]. In electrochemical [11] and environmental fields [12], nano-MnO_2_ is a novel material that increases the exposure to Mn during production and application. Due to the potential application in biomedical imaging fields [13,14], the exposure risk of human beings to manganese-based magnetic nanoparticles also increased. In medical applications, these nanoparticles can be injected *in vivo*. Further, nanomaterials can enter the body via the respiratory tract; for example, welding fumes generally contain Mn metal and manganese oxide particles, therefore, welders have high exposure risk [15], and the deposition of Mn in the brain areas of welders is extensive enough to produce a magnetic resonance imaging (MRI) response [16]. These materials can also enter the body by gastrointestinal absorption and dermal contact; some nanomaterials enter the central nervous system [17] via the bloodstream or nasal olfactory nerve endings. It is known that increased manganese (Mn) exposure causes neurotoxic effects, like cognitive, psychiatric and motor deficits *in vivo* [16]. The long-term and low-dose Mn exposure induced dopaminergic impairment [18], this condition may be associated with intracellular oxidative stress, mitochondrial damage, and protein aggregation [19], and alteration of glia components in brain [20]. Studies also showed that Mn poisoning exhibits similar molecular alterations, motor behaviors, and nervous system symptoms to Parkinson’s syndrome at early stages [21].With the increasing exposure potential, neurotoxicology of nano-MnO_2_ should be assessed in terms of bio-security.

In the present experiment, nano-MnO_2_ was injected in the rat brain with the aid of a stereotaxic instrument to investigate the neurotoxicity of nano-MnO_2_. 6-Hydroxydopamine (6-OHDA) was used as a positive control treatment and saline was administered as a negative control treatment. Nano-MnO_2_-induced behavioral effects on rats were investigated using Morris water maze experiments. Changes in tyrosine hydroxylase (TH) and glial fibrillary acidic protein (GFAP) expressions were analyzed by immunohistochemistry.

## 2. Experimental Section

### 2.1. Reagents and Instruments

The following substances were used in this study: nano-MnO_2_ (diameter = 10 nm; concentration = 87 μg/μL; Amresco, Solon, OH, USA); 6-OHDA; apomorphine (APO), mouse anti-rat TH monoclonal antibody and polyclonal rabbit anti-rat GFAP antibody (Sigma, Saint Louis, MO, USA). General-purpose chain enzyme peroxidase-labeled avidin (SP-9000) immunohistochemistry kit (Beijing Zhongshan Golden Bridge Biotech, Beijing, China) and concentrated DAB color kit (ZLI-9032; Beijing Zhongshan Golden Bridge Biotech) were also used.

The following instruments and systems were also used: stereotaxic apparatus (Jiangwan II, Shanghai Second Military Medical University, Shanghai, China); Morris water maze and video systems RD1101-MM (Shanghai Yishu Info. Tech., Shanghai, China); freezing microtome (CM-1900, Leica, Wetzlar, Germany); optical microscope (Olympus, Nagano, Japan); and image processing system (Leica).

### 2.2. Experimental Designs

Forty rats were screened by a training phase of Morris water maze test, then the two rats which had extremely short and the two which had extremely long escape latencies were removed. The remaining 36 rats were randomly grouped (12 rats per group) as nano-MnO_2_-, 6-OHDA-, and saline-treated groups. Following the intracerebral injection, Rotation behaviors of rats were detected. 7 and 14 days after injection, Morris water maze test were performed to observe the cognitive capability of rats (12 rats per group on 7 day, 8 rats per group on 14 day). 7, 14 and 28 days after injection, the expression of TH, GFAP and iNOS were detected in rats brain (4 rats per group for each time point) tissue by immunohistochemistry.

### 2.3. Animal Experiments

Male Sprague-Dawley rats (200 g to 250 g; Experimental Animal Center of Southeast University) were housed with equal daily periods of light and dark cycles and provided free access to food and water. The animal protocols used in this work were conducted in accordance with the Declaration of Helsinkin, and approved by Ethics Committee of Southeast University (Project identification No. 201105012).

The rats were anesthetized with 0.4% sodium pentobarbital (40 mg/kg body wt., i.p.), fixed in the stereotaxic apparatus, and subjected to conventional disinfection and craniotomy. The bregma was exposed by creating a top middle skin incision. The injection targets were the substantia nigra pars compact (SNc) and the ventral tegmental area (VTA), which were identified according to Paxinos-Watson atlas of rat [22]. The SNc injection point coordinates (mm) were as follows: AP, −4.8; ML, 1.8; DV, 9.2. The VTA injection point coordinates (mm) were as follows: AP, −4.8; ML, 1.0; and DV, 9.2. Approximately 1 μL of 87 μg/μL nano-MnO_2_, 1 μL of 9 μg/μL 6-OHDA solution, or 1 μL of solvent (0.9% NaCl) was injected at a rate of 0.5 μL/min using a 5 μL microsyringe. The needle was retained for 5 min after injection, withdrawn 0.5 mm, then the needle was retained at this position for another 5 min, withdrawn slowly at a rate of 2.0 mm/min. Afterward, the rats were kept warm and fed when they were conscious.

### 2.4. Rotation Behavior

The severity of dopamine depletions was evaluated by assessments of rotational behavior. To measure nanoparticles induced behavioural lesion, the animals were injected with 0.05 mg/kg APO one week after exposure. The number of rotations ipsilateral and contralateral to the lesion during a 40-min interval after APO injection was recorded.

### 2.5. Morris Water Maze Test

A Morris water maze test was performed before injection as training phase. The diameters of the water maze and the platform were 160 and 11 cm, respectively. The platform was placed at a fixed location in the center of one of the four imaginary quadrants. Three starting points were set at the midpoint in round basin of the imaginary quadrants except the one where the platform was located. Twenty mL of ink was added to the water to form an opaque solution at 25 ± 1 °C. Visual cues outside the water maze were relatively fixed. For the training experiment, the rats were released at one of the three different starting positions. The rats were allowed to spend 180 s to find the hidden escape platform in water and stayed there for 10 s. If the rats could not find the platform within 180 s, the experimenter helped them locate this platform and the rats stayed there for 10 s. Continuous training was performed four times a day for 4 days and 24 h after the last training session, rats were injected. Seven days and 14 days after injection, the rats were released from one of the starting positions, which was opposite to the quadrants where the platform was, four times and allowed to swim for up to 120 s. Distance travelled, swim speed and escape latency were measured in each trial.

### 2.6. Immunohistochemical Staining

After the Morris water maze test was conducted, the rats were anesthetized with 0.4% sodium pentobarbital (40 mg/kg body wt., i.p.) and perfused intracardially with 400 mL of 4% paraformaldehyde. The brains were removed and post-fixed in 4% paraformaldehyde for 24 h and stored in 30% sucrose solution. The middle brain was obtained for continuous coronal slicing (30 μm). Immunohistochemical staining steps were summarized as follows: (1) the slices were cooled to room temperature, washed with PBS (pH 7.4; 0.01 mol/L) twice for 5 min, and incubated in 3% H_2_O_2_ for 15 min; (2) the slices were washed thrice with PBS for 5 min; 10% goat serum working solution was then added at 37 °C for 30 min without washing; (3) anti-TH (1:10,000), iNOS(1:100) and GFAP (1:80) were added at 4 °C and stored overnight; (4) the slices were washed with PBS thrice for 5 min; biotin-labeled secondary antibody working solution was added at 37 °C for 60 min; (5) the slices were washed again with PBS thrice for 5 min; alkaline phosphatase-labeled streptavidin was added at 37 °C for 30 min; (6) the slices were washed again with PBS thrice for 5 min and stained with DAB; double-distilled water was used to terminate the reactions; (7) PBS was placed in the patch, dehydrated, transparent, and mounted. Another slice was used as a negative control sample, in which normal goat serum was used instead of the primary antibody.

After immunostaining, the brain sections were examined under light microscopy by two histologists blinded to treatment conditions. The immunoreactivities of TH, GFAP and iNOS were evaluated. The number of TH^+^, GFAP^+^ and iNOS^+^ cells of each rat brain section was counted in three nonoverlapping high-power fields (HPFs; 400× magnification) and analyzed. The HPFs were selected from hippocampal areas for GFAP, SNc and VTA regions for TH and iNOS that had a maximum of positive cells. In each studied field, only positive cells with the nucleus at the focal plane were counted.

### 2.7. Statistical Analysis

Indicators of the Morris water maze tests were analyzed by ANOVA, followed by student’s *t* test for parametric comparison between groups. The immunostaining results were statistically analyzed by ANOVA, followed by Student’s *t* test or nonparametric *t* test statistics. Results are shown as arithmetic means and standard errors of means. SPSS 13.0 software was used for statistical analysis, and the significance level was set at *p* < 0.05.

## 3. Results and Discussion

### 3.1. Motor Behavioral Study

The rats showed *in situ* rotation from head to tail with center of the injection-contralateral hindlimb one week after exposure and 5 min after APO injection in 6-OHDA treatment group. In the nano-MnO_2_ group, no head-to-tail rotation was observed after injection, but abnormal behaviors, such as tremors, slow activity, sniffing, irritability and vertical tail, were shown. Mn dust exposure also induces these Parkinson-like symptoms [19]. Our result suggested a slight extrapyramidal impairment, but the typical Parkinson’s symptom, which is the head-to-tail rotation, was not observed.

### 3.2. Morris Water Maze Test

Morris water maze test results are shown in Table 1. The escape latencies of nano-MnO_2_- and 6-OHDA-treated rats showed a significant increase compared with those in the saline group (* *p* < 0.05, ** *p* < 0.01) both 7 and 14 days after injection, however, the escape latencies in the 6-OHDA group was significantly longer than that in nano-MnO_2_-treated group 14 days after injection. The velocity of 6-OHDA-treated rats also showed a significant decrease compared with the saline group (* *p* < 0.05).

Mn exposure showed adverse effects on cognition capability. In rodents, the hippocampus is a structure responsible for spatial navigation [23,24]. Hippocampal place cells are orientation-invariant and location-specific, and are mainly located in CA1 and CA3 region of hippocampus [24,25]. Two weeks of intranasal manganese chloride exposure induced spatial memory deficits in rats [26]. High-manganese diet also results in behavioral deficits of rats in the Morris water maze tests [27]. Our results suggested that nano-MnO_2_ impair the spatial learning of rats, while when compared with the positive control, 6-OHDA, showed less toxic effects. Other nanoparticles may affect neurobehaviors once the central nervous system is contaminated. Zinc oxide nanoparticles extend the escape latency of mice in the Morris water maze test [28]. Maternal exposure to titanium dioxide nanoparticles during lactation period can also cause impairments on learning and memory ability of rat offspring [29]. These cognitive impairments are associated with damage to the hippocampus.

**Table 1:** Escape latency and score of rats in Morris water maze test (*x* ± s).

	7 Days after Injection			14 Days after Injection		
	Saline	6-OHDA	Nano-MnO_2_	Saline	6-OHDA	Nano-MnO_2_
Escape latencies (s)	27.62 ± 28.73	64.68 ± 43.62 **	63.89 ± 42.73 **	25.12 ± 29.03	67.69 ± 51.61 **	60.52 ± 41.24 ^##,^**
Velocity (cm/s)	24.81 ± 4.35	19.28 ± 3.77 *	21.59 ± 3.61	23.75±3.69	18.72 ± 4.65 *	21.64 ± 3.32

^#^ *p* < 0.05, ^##^* p* < 0.01, compared with 6-OHDA group; * *p* < 0.05, ** *p* < 0.01, compared with saline group. For the 7 days after injection Morris water maze test, *n* = 12; 14 days after injection Morris water maze test, *n* = 8.

### 3.3. Immunohistochemical Staining

**Figure 1:**
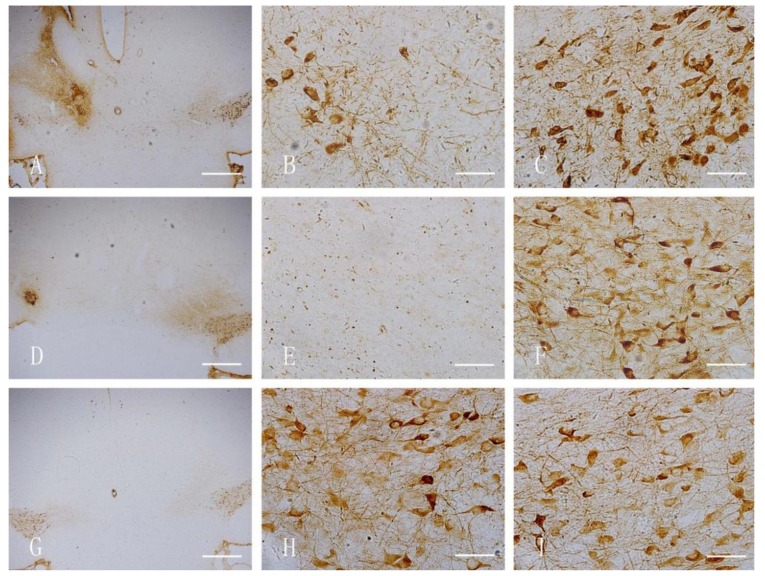
TH immunohistochemical staining of rat midbrain. (**A**) Damaged side of nano-MnO_2_ group (40×), Bar = 500 μm; (**B**) damaged side of nano-MnO_2_ group (400×), Bar = 50 μm; (**C**) uninjured side of nano-MnO_2_ group (400×), Bar = 50 μm; (**D**) damaged side of 6-OHDA group (40×), Bar = 500 μm; (**E**) damaged side of 6-OHDA group (400×), Bar = 50 μm; (**F**) uninjured side of 6-OHDA group (400×), Bar = 50 μm; (**G**) damaged side of saline group (40×), Bar = 500 μm; H: damaged side of saline group (400×), Bar = 50 μm; and I: uninjured side of saline group (400×), Bar = 50 μm.

**Figure 2:**
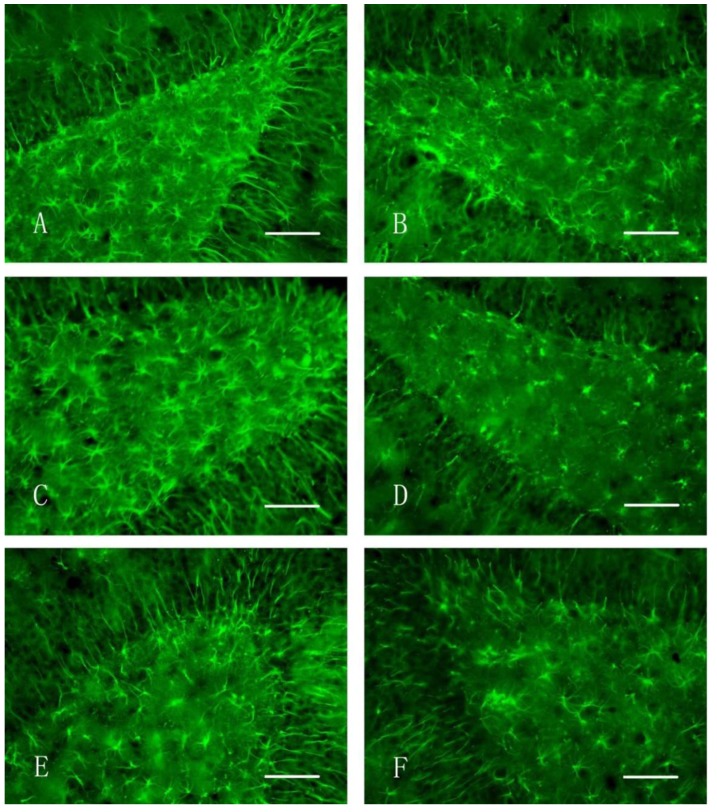
GFAP immunohistochemical staining of the hippocampus of rats (400×, Bar = 50 μm). (**A**) Damaged side, nano-MnO_2_ group; (**B**) uninjured side, nano-MnO_2_ group; (**C**) damaged side, 6-OHDA group; (**D**) uninjured side, 6-OHDA group; (**E**) damaged side, saline group; and (**F**) uninjured side, saline group.

**Figure 3:**
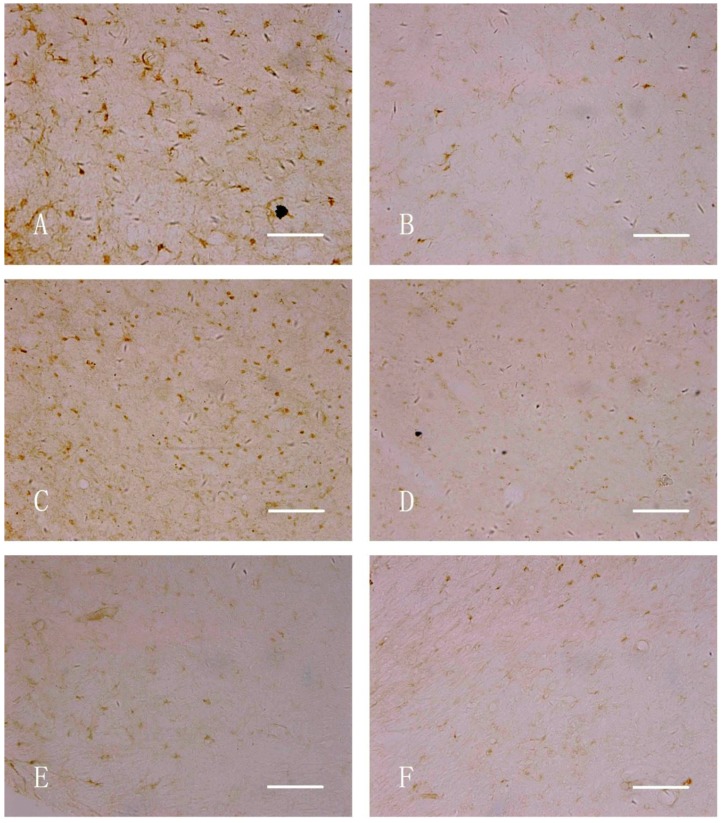
iNOS immunohistochemical staining of the midbrain of rats (400×, Bar = 50 μm). (**A**) Damaged side, nano-MnO_2_ group; (**B**) uninjured side, nano-MnO_2_ group; (**C**) damaged side, 6-OHDA group; (**D**) uninjured side, 6-OHDA group; (**E**) damaged side, saline group; and (**F**) uninjured side, saline group.

**Figure 4:**
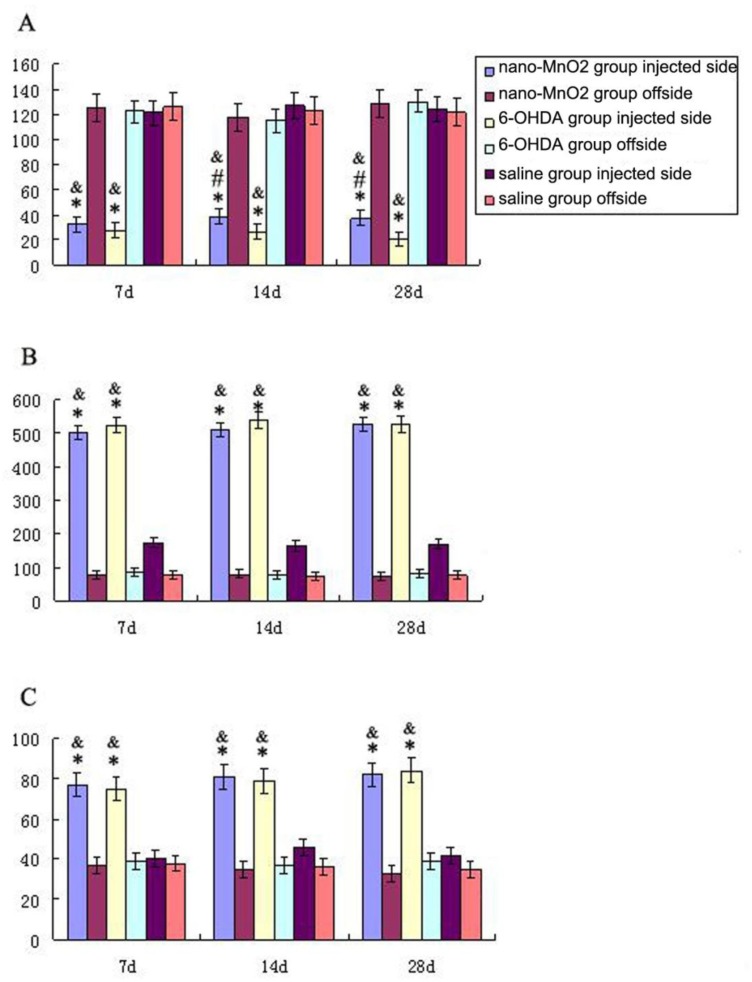
Number of immunostained positive cells in both sides of rat brain. (**A**) TH-positive cells, (**B**) GFAP-positive cells; and (**C**) iNOS-positive cells (* *p* < 0.05 compared with saline injected side, ^#^ *p* < 0.05 compared with 6-OHDA injected side, ^&^ *p* < 0.05 compared with contralateral).

Figure 1 shows the TH-stained rat brains. In both the nano-MnO_2_ and 6-OHDA groups, TH-positive cells on the damaged sides (B, E) were significantly less than those of the contralaterals (C, F) and the damaged side of the saline group (H) at 7 d after injection. Figure 2 shows the GFAP-stained hippocampus of the rats. In both the nano-MnO_2_ and 6-OHDA groups, GFAP-positive cells on the damaged side (A, C) were significantly higher than those of the contralaterals (B, D) and the damaged side of the saline control (E, F) at 7 days after exposure. Figure 3 also shows the rat brains subjected to iNOS immunohistochemical staining. In nano-MnO_2_ and 6-OHDA groups, iNOS-positive cells on damaged sides (A, C) were significantly higher than those of the contralaterals (B, D) and damaged sides of the saline group (E) one week after injection. The number of TH-, GFAP-, and iNOS-positive cells were counted for 10 non-overlapping random views (Figure 4). After 7, 14, and 28 d of injection, the number of TH-positive cells decreased, but the number of GFAP- and iNOS-positive cells increased significantly compared with that in the contralateral brain and the saline-treated lateral brain.

Dopamine (DA) is an important central neurotransmitter involved in the regulation of neuropsychiatric activities. The central substantia nigra DA system is the major center of the extrapyramidal system of motor functions. DA dysfunction can lead to serious neurological and psychiatric disorders, thereby causing behavioral changes. Mn-nanoparticles induce apoptosis in dopaminergic neuronal cells *in vitro* [30] and may involve mechanisms associated with neurodegeneration [31]. Other nanomaterials, such as silica, can decrease dopamine concentrations in the striatum and impair dopaminergic neurons [32]. In another study, dopamine-related gene expression is altered in cultured PC12 cells after exposure to Ag and Cu nanoparticles [31]. Our results showed that TH is expressed at a less extent in the nano-MnO_2_-injected side of the midbrain than in the saline-treated contralateral, which is consistent with the *in vitro* tests of Mn-nanoparticles. It is indicated that Mn-nanoparticles induced dopaminergic neuronal impairment *in vivo* and this can explain the changes in the motor behavior of rats, however, when compared with the positive control, 6-OHDA injected rats, the dopaminergic neuron dysfunction is still at early stage, for no head-to tail rotation is observed.

iNOS expression in the damaged sides is significantly higher than that in the contralaterals. This result may be associated with inflammatory reaction after nano-MnO_2_ stimulation. Inflammation is one of the major bioeffects of nanomaterials in organisms [33,34,35]. The astrocytes derived iNOS can synthesize large amounts of NO and on a slower time scale [36]. NO is secreted as free radicals and is a critical mediator of inflammation in pathological processes [37]. Excessive NO mediates neurological toxicity and cytotoxicity, thereby causing tissue damage; furthermore, excessive NO directly or indirectly induces neuronal impairment [38].

GFAP is the main component of the collagen in an astrocyte body; GFAP is slightly or negatively expressed in a normal brain. Astrocytes are rapidly transformed to an activated state when the brain tissue is damaged; as a result, the number of cells and GFAP expression are significantly increased. Activated astrocytes then function to protect and induce toxicity on neurons; this dual function is called a double-edged effect [39]. GFAP expression in the damaged side of the hippocampus was upregulated significantly compared with that in the contralateral and damaged side of the saline-treated rats. This result indicated that nano-MnO_2_ increased astrocyte activity. Changes in the inner environments of the hippocampus may also affect the memory ability of nano-MnO_2_-injected rats.

## 4. Conclusions

In summary, our results showed that nano-MnO_2_ injected intracerebrally in the rat brain induced changes in the locomotor and spatial memory abilities of rats, which is associated with dopaminergic neuronal impairment and MnO_2_ nanoparticle-induced inflammation. When compared with the positive control, 6-OHDA, MnO_2_ nanoparticles only induced the early stage symptoms of extrapyramidal impairment with a selective loss of dopaminergic neurons, however, considering the potential use of MnO_2_ nanoparticles in diagnosis or treatment procedures, a proper dose should be carefully selected to avoid potential neurotoxic effects.
